# Enabling Action: Reflections upon Inclusive Participatory Research on Health with Women with Disabilities in the Philippines

**DOI:** 10.1002/ajcp.12468

**Published:** 2020-10-20

**Authors:** Cathy Vaughan, Liz Gill‐Atkinson, Alexandra Devine, Jerome Zayas, Raquel Ignacio, Joy Garcia, Krissy Bisda, Joy Salgado, M. Jesusa Marco

**Affiliations:** ^1^ Centre for Health Equity The University of Melbourne Melbourne Vic. Australia; ^2^ Nossal Institute for Global Health The University of Melbourne Melbourne Vic. Australia; ^3^ Social Development Research Center De La Salle University Manila Philippines; ^4^ Center for Women’s Studies Foundation University of the Philippines Quezon City Philippines; ^5^ WOWLEAP Cainta Philippines; ^6^ PARE Quezon City Philippines; ^7^ Likhaan Center for Women’s Health Quezon City Philippines

**Keywords:** Community‐based participatory research, Disability, Inclusion, Sexual and reproductive health, Violence, Solidarity, Philippines

## Abstract

People with disabilities experience health disparities arising from social, environmental, and system‐level factors. Evidence from a range of settings suggests women with disabilities have reduced access to health information and experience barriers to screening, prevention, and care services. This results in greater unmet health needs, particularly in relation to sexual and reproductive health. Women with disabilities are also more likely to experience physical and sexual violence than women without disabilities, further undermining their health. Community‐based participatory research (CBPR) can generate knowledge and underpin action to address such health disparities and promote health equity. However, the potential and challenges of disability inclusion in CBPR, particularly in contexts of poverty and structural inequality such as those found in low‐ and middle‐income countries, are not well documented. In this paper, we reflect on our experience of implementing and evaluating W‐DARE, a three‐year program of disability‐inclusive CBPR aiming to increase access to sexual and reproductive health and violence‐response services for women with disabilities in the Philippines. We discuss strategies for increasing disability inclusion in research and use a framework of reflexive solidarity to consider the uneven distribution of the benefits, costs, and responsibilities for action arising from the W‐DARE program.

## Highlights

- Disability inclusive community‐based participatory research can address health inequalities.
- Inclusion increases quality and impact of research, but has material, personal and political costs.
- The costs of inclusive research are disproportionately born by co‐researchers with disabilities.
- Participatory research can contribute to new solidarities for sustained health‐promoting action.

## Introduction

People with disabilities across all settings experience significant health disparities. The United Nations Convention on the Rights of Persons with Disabilities (CRPD) upholds the rights of people with disabilities to access appropriate health information and services on an equal basis with others (United Nations, 2006). However, the limited capability of health systems to be responsive and inclusive of people with disabilities undermines implementation of the CRPD in many places. This is particularly the case in low‐ and middle‐income countries (LMICs; MacLachlan & Swartz, 2009). While people with disabilities have the same and often additional healthcare needs as people without disabilities, in most places, people with disabilities report a range of factors undermining their ability to access health services (WHO & the World Bank, 2011). People with disabilities often have limited awareness of their own health rights and this further undermines their access to appropriate health information and services (MacLachlan & Swartz, 2009). Unique and compounded disadvantage relating to gender inequality and disability discrimination often means women with disabilities experience greater barriers to accessing health services and experience poorer health outcomes, particularly in relation to sexual and reproductive health (SRH), compared to women without disabilities and men with disabilities in their communities (Dowse, Frohmader, & Didi, 2016). As one in seven people worldwide has a disability and more than one in five women in LMICs has a disability, this is a significant global issue (WHO & the World Bank, 2011).

For many decades, researchers have drawn on community‐based participatory research (CBPR) approaches to actively involve people in research to address the health disparities they face. CBPR approaches promote health equity by creating new knowledge about the health issues and concerns facing marginalized communities and through the direct participation of community members. A substantial and growing body of evidence confirms that CBPRapproaches can improve health disparities for some marginalized individuals and groups through behavior change, advocacy, and activism for structural change (Wallerstein, Duran, Oetzel, & Minkler, 2017). However, there is less evidence examining the intersection of CBPR approaches and disability.

Disability‐inclusive research approaches, informed by the work of disability activists in the 1960s, have transformed how people with disabilities are engaged by researchers in many places (Barnes, 2003). Historically, people with disabilities have been subjected to extractive and sometimes exploitative research practices by researchers who conducted research “on” not “with” people with disabilities (Barton, 2005). Disability‐inclusive approaches have resulted in increased awareness of the importance and benefits of actively involving people with disabilities in research as collaborators, not only as research subjects. In the early 2000s, advocacy for and the introduction of the UNCRPD increased awareness of the rights of people with disabilities to be actively involved in research and development programming, leading to the increased popularity of disability‐inclusive research and development approaches in LMICs (Grech, 2016). However, documentation of efforts to ensure CBPR is inclusive of people with disabilities is limited. Many people with disabilities in LMICs, and in particular women with disabilities, experience substantial barriers to active and meaningful involvement in research and subsequent development programming. However, critical reflection about how a CBPR to research can be inclusive of women with disabilities in LMICs, and about what is required for inclusion in research in complex contexts of prejudice, poverty, and inequality, is limited in the published literature.

To contribute toward efforts to redress this knowledge gap, in this paper we draw on our experience with the Women with Disabilities taking Action on REproductive and sexual health program (W‐DARE), a three‐year program of disability‐inclusive, CBPR in the Philippines. In the Philippines, approximately 12 percent of the population experience significant disability (Philippines Statistics Office, 2019), and the country ratified the CRPD in 2008. The Philippines has enacted several laws and policies to promote the rights of people with disabilities; however, these are not always understood or enforced, with people with disabilities in the Philippines facing discrimination in a range of settings and numerous barriers to services (Jandayan, Figueroa, & Canales, 2009).

W‐DARE aimed to increase access to SRH services, including violence‐response services, for women with disabilities. Our analysis of this experience is underpinned by a theoretical framework of reflexive solidarity (Davis & Vaughan, 2018). Reflexive solidarity draws from and brings together two well‐established features of CBPR; reflexivity and solidarity. Reflexivity has been defined as “the ability to reflect inward toward oneself as an inquirer; outward to the cultural, historical, linguistic, political, and other forces that shape everything about inquiry; and, in between researcher and participant, to the social interaction they share” (Sandelowski & Barroso, 2002, p. 212). Solidarity has been defined as “an enacted commitment to carry ‘costs’ (financial, social, emotional or otherwise) to assist others with whom a person or persons recognize similarity in a relevant respect” (Prainsack & Buyx, 2012, p. 52). In the context of CBPR, reflexive solidarity involves recognizing the “similarity in a relevant respect” of people collaborating on research—a shared desire to generate knowledge for action—but also recognition that people come to the collaboration with different privileges and opportunities and bear the costs of their engagement in very different ways (Davis & Vaughan, 2018).

CBPR has enormous potential to generate knowledge and action that can contribute to health equity and healthy communities (Wallerstein et al., 2017). However, community‐based participatory approaches also have the potential to benefit some community members while excluding others; to reinforce inequalities, discrimination, and prejudice within communities; and to lead to unanticipated burdens for some. A framework of reflexive solidarity supports critical reflection upon the distribution of costs, benefits, and responsibilities for action among the diverse actors involved in CBPR projects (Vaughan et al., 2019).

The program of research we are drawing on as a case study in this paper, W‐DARE, was a complex project involving individuals and organizations with wildly divergent access to power and resources; who were geographically, politically, socially, culturally, and religiously disparate; and who brought to the program different histories of privilege, prejudice, and struggle. This complexity is common in the messy business of CBPR, and therefore, our reflections have relevance for efforts toward inclusion and equity beyond the Philippines.

## Processes and Data

W‐DARE was a three‐year program of disability‐inclusive, CBPR implemented between 2013 and 2016 in two research sites in the Philippines: Quezon City in Metro Manila and Ligao City in Albay Province. The aim of the W‐DARE program was to improve the SRH of women with disabilities in the Philippines by identifying the factors that influence their access to SRH information and services; developing, implementing, and evaluating local interventions to address these factors; and developing and disseminating guidelines to inform provision of gender‐sensitive, disability‐inclusive SRH and violence‐response services in the Philippines (Vaughan et al., 2015). The research was funded by the Australian Department of Foreign Affairs and Trade and the UNFPA United Nations Fund for Population Activities (UNFPA) Philippines country office. It received ethics clearance from the University of Melbourne Human Research Ethics Committee (in Australia) and the De La Salle University Ethics Committee (in the Philippines).

W‐DARE was led by researchers at the University of Melbourne (Australia) and De La Salle University (Philippines) in partnership with national Disabled People’s Organizations (DPOs) WOWLEAP and PARE, the national non‐profit SRH service provider Likhaan Center for Women’s Health, and the Center for Women’s Studies Foundation (University of the Philippines). DPO representatives (who were women with disabilities) were actively involved in identifying the research problem (including collaborating on a UNFPA‐funded pilot study that informed W‐DARE), designing the research, recruiting co‐researchers, making decisions about the research implementation, and the conduct of research activities (data collection, analysis, and dissemination). Co‐researcher positions were advertised in the community through the DPOs, and women with mobility impairments, women with vision impairments, and women who were deaf were recruited as co‐researchers and provided with extensive training to undertake data collection and analysis. This included working with research team members from the other partner organizations to conduct the Rapid Assessment of Disability (RAD) quantitative household survey to establish the prevalence of disability in the two research sites (Marella et al., 2016); conducting face‐to‐face qualitative interviews with other women and girls with disabilities (Vaughan et al., 2016); facilitating peer‐supported Participatory Action Groups with women with disabilities and parents of children with disabilities (Devine et al., 2017); and co‐facilitating interventions with local health and violence‐response service providers and policy makers (Vaughan et al., 2016).

To present a holistic view of the W‐DARE program, and discuss the effectiveness of CBPR to address health disparities and promote health equity for women with disabilities in the Philippines, we have drawn from a wide range of data sources. These include published peer‐reviewed journal articles about the W‐DARE program; reports and case studies submitted to the program’s donors; articles published on the public W‐DARE blog; and dialogical interviews conducted over Skype between Australian and Filipino researchers in mid‐2019. They also include transcripts of face‐to‐face in‐depth interviews conducted by author 2 as part of her PhD research examining empowerment and participatory research. These 57 interviews were conducted between 2014 and 2016 with the co‐researchers and other Filipino research team members. Table 1 summarizes the different stages of the W‐DARE program, aligning key research activities with the data sources subsequently available for inclusion in our analysis for this reflective paper.

**Table 1:** Relationship between W‐DARE activities and data sources for this reflective paper

Stage	Major activities	Data available for reflection
Design (2012)	Design of program and application for funding	Pilot research report Research proposal
Research phase 1 (2013–2014)	Identification of factors influencing access to SRH and violence‐response information and services through Co‐researcher recruitment and training RAD household survey in‐depth interviews and focus group discussions with women with disabilities, service providers, and families	Peer‐reviewed papers (Lee et al., 2015; Marella et al., 2016; Vaughan et al., 2015, 2016) Transcripts from interviews with research team members, including co‐researchers (19) Community report
Research phase 2 (2014–2016)	Development, implementation and evaluation of pilot interventions to increase access to SRH and violence‐response services Supply side: Sensitisation workshops for service providers; health facility audits; health worker training Demand side: Participatory Action Groups (peer support groups) Enabling environments: exchange visits, stakeholder engagement, advocacy videos	Peer‐reviewed papers (Carroll et al., 2016; Devine et al., 2017) Evaluation reports (2) Service provider training package Reports to program donors (3) Case studies for donor (2) Blog posts (16) Transcripts from interviews with research team members, including co‐researchers (38)
Research phase 3 (2016)	Development and dissemination of guidelines and tools to inform provision of disability‐inclusive SRH and violence‐response services	Guidelines and fact sheets PAG facilitator manual RAD toolkit Policy briefs (2)
Post program reflection (2019)	Recorded conversations between partners and academics in the Philippines and Australia, reflecting on program impact and the experience of inclusive research, 3 years after W‐DARE formally ended	Transcripts and notes from dialogical interviews involving six research team members (3)

We took a narrative approach to analysis of this large collection of data, to examine whether and how program activities were inclusive of women with disabilities in W‐DARE, and how inclusion was supported. Drawing on Greenhalgh, Russell, and Swinglehurst (2005) description of the use of narrative for collective sense making, in our dialogical interviews in 2019 we jointly reflected upon the volume of program documentation to consider how the experience and impacts of this inclusion were discussed and written about throughout the life of the program, and by whom. This enabled us to synthesize diverse perspectives on the emergent “story” of W‐DARE as an example of disability‐inclusive CBPR. We used the theoretical framework of reflexive solidarity (Davis & Vaughan, 2018) to identify benefits and costs of a disability‐inclusive approach to CBPR, and how these were distributed across all actors involved. By combining a narrative approach with this theoretical framework, we were able to critically reflect upon how the costs and benefits of participation were made visible or hidden in the story of W‐DARE presented in program reports, academic papers, and public documents.

Our analysis of the comprehensive collection of W‐DARE data identified that a disability‐inclusive approach to research contributed to the quality and impact of the project, and to the capacity and commitment of the team. However, we also identified that the distribution and sustainability of the benefits associated with such an approach were uneven, as was the distribution of costs.

## Reflections on our Experiences

Women with disabilities were centrally involved in decision‐making about W‐DARE before the program even began. Women with disabilities, through their representative organizations, had expressed the need to better understand and address barriers to SRH for women with disabilities in the Philippines. UNFPA responded to this expressed need by supporting pilot research, undertaken by a Filipino team consisting of women with disabilities (including authors 6 and 7) working with a local consultant (author 4) to summarize the relevant policy and legislative landscape and to synthesize the existing research evidence. UNFPA had also been in contact with Australian researchers (authors 1 and 3) about the need for evidence that could be used to increase access to SRH and violence‐response services in the Philippines. Shortly thereafter, an appropriate funding opportunity was identified, and a collaborative international team formed in order to develop and submit a proposal. The women’s DPOs that had been involved in the pilot research were key members of the collaboration developing this proposal. Our application was successful. The circumstances under which W‐DARE began—where a group had expressed a clear health research need, were connected to researchers with relevant skills, and then substantive funding became available—are important to highlight, as such serendipitous events do not often occur. It is also important to highlight that inclusion of women with disabilities from the beginning meant that the research questions being addressed were relevant to and a priority for women with disabilities.

### Inclusion Contributes to the Quality and Impact of Research

The fact that the design of W‐DARE data collection tools and processes was inclusive of women with disabilities resulted in a number of clear benefits. Women with disabilities were part of the enumerator team for the household survey and were able to inform the survey team about their community from the perspective of a person with disability, reducing barriers to respondents. This was the first time that a stand‐alone disability prevalence survey had been undertaken in the Philippines, producing data that could be used to underpin planning, policy, and service delivery across a range of sectors (Marella et al., 2016). Input from co‐researchers helped ensure qualitative interview questions and processes enabled women with disabilities to talk about highly sensitive topics such as SRH and violence. Co‐researchers alerted the team to emergent safety risks (e.g., when communicating in sign language) to reduce the possibility the research might inadvertently cause harm. Our disability‐inclusive approach also led to the generation of rich, new information about the intersecting factors that undermine the SRH of women with disabilities in the Philippines, including their experiences of violence. As one of the Filipino researchers noted, women with disabilities collecting data “improves the process, because we get more insightful and accurate information… the data becomes much more valid” and “it would not have been possible to collect the information otherwise.”

As a CBPR project, it was always our intention that analysis of data collected early in the project would underpin design of pilot interventions to address barriers to SRH that we identified. It was very clear that barriers to SRH for women with disabilities were on both the “supply” and “demand” side of SRH services; however, during analysis workshops the co‐researchers pointed out that these barriers were shaped by local environments and were situated in an over‐arching societal context of negative attitudes toward people with disabilities. As a result, we designed and piloted interventions to address barriers in all these domains as shown in Table 1.

On the “supply” side, we ran sensitization sessions for health workers to address service providers’ lack of knowledge and skills in relation to disability, as well as prejudice and discrimination. We conducted accessibility audits of health facilities, where women with different types of impairment would “walk through” the service with facility managers and service providers to identify barriers to access. We also conducted specific training of doctors in relation to SRH and disability. Evaluation of the trainings and other activities undertaken as part of the cycle of action research in W‐DARE, suggested that it was the contribution of women with disabilities to these activities that had the most impact on service providers’ knowledge and attitudes. As one of the co‐researchers noted:

It is more effective and there is more impact if the person with disability is the one giving the orientation and being the one doing the training… Because not only did the [health service providers] come to realize about their [the women’s] ability, but also that their hospital needs to be accessible. Not only the hospital, the government units, the local government agencies that has offices that are not accessible. This has more impact… because if you are not a person with disability you do not see and realize how difficult it is.

Following the training and sensitization of health workers, DPO representatives were regularly included in local and national consultations about women’s health policy and practice, and there were strengthened relationships between DPOs and relevant government departments. Health service providers reported increased capacity to communicate with and provide disability‐inclusive services to women with disabilities. Service providers also made concrete changes to increase the physical accessibility of their facilities, began to provide outreach services, and reported increased recognition of the SRH rights of women with disabilities. These changes resulted in increased access to services for women with disabilities in Quezon City and Ligao City.

Analysis of program data also informed the design of activities to address the “demand” for SRH services. Our analysis suggested that many women with disabilities had little knowledge about SRH and their rights, poor mental health, limited awareness of services, and a fear of health services. Therefore, we developed an intervention‐based around peer‐facilitated Participatory Action Groups (PAGs), where we trained co‐researchers and other women with disabilities to facilitate groups of their peers to share information and discuss and debate SRH and related issues. This intervention was developed in close partnership with local SRH service providers, ensuring the accuracy of information discussed. The partnership strengthened women’s links with and trust in local services, and increased the awareness and confidence of service providers in relation to disability. Evaluation of the PAGs found participants groups reported increased knowledge on SRH, and increased confidence and peer support to access services (Devine et al., 2017).

Interventions to foster change in local environments prioritized sensitization of city‐level government representatives, with exchange visits between the two W‐DARE sites fostering learning about both disability and SRH service delivery. Team members engaged with district and *barangay* (the smallest unit of administration in the Philippines) level representatives to build ongoing support for women with disabilities in their communities. The program also developed videos that focused on the capacities and strengths of women with disabilities, rather than the stereotypical deficit‐based representation of disability common in the Philippines. These videos continue to be used in advocacy and other activities long after the program’s formal completion, highlighting the ways in which non‐traditional research outputs can have specific benefits to communities in ways that more traditional products (such as journal articles) may not.

### Inclusion Contributes to Team Capacity and Commitment

The central, visible, and active participation of women with disabilities in the conduct of research activities throughout the program was profoundly impactful on both these women and on the Australian and Filipino academics, activists and service providers they were working alongside. One of the benefits most commonly reported by team members was their increased technical capacity to conduct disability‐inclusive research, with this new expertise being recognized by national and international stakeholders. Members of the research team (Filipino academics and co‐researchers) were contracted to conduct disability‐inclusive research following Typhoon Haiyan. They have also subsequently been contracted by government to advise on the provision of disability‐inclusive health services and public transport, and have conducted training on disability‐inclusive development and the collection of disability prevalence data for international funding bodies. Alongside the development of technical research skills, team members and particularly women with disabilities demonstrated increased skills in advocacy. Women with disabilities involved in W‐DARE have spoken out publicly about the need for SRH services to be disability‐inclusive. In particular, they have made important contributions to strengthening disability inclusion in the implementation of the national Responsible Parenthood and Reproductive Health Act of 2012, known throughout the country as the RH Law. This legislation mandates access to SRH information and services across the Philippines. Through meetings with the National Implementation Team guiding roll out of the law, women with disabilities have been able to advocate for their rights and needs, and disseminate a W‐DARE policy to practice brief specifically focused on disability inclusion and the RH Law.

Women with disabilities, both co‐researchers and PAG participants, describe their involvement in W‐DARE as substantially expanding their networks, leading to a range of new opportunities. Co‐researchers and PAG participants have been invited to speak at national conferences, to testify before the Philippines Commission on Human Rights, to travel overseas to represent the Philippines at Convention on the Elimination of All forms of Discrimination Against Women (CEDAW) working meetings on violence against women, and to present at international conferences. Some of the women taking up these opportunities were significantly socially isolated prior to their involvement in W‐DARE, and experienced them as highly transformative of both their confidence and life chances.

Other team members described transformative change in their own attitudes. One Filipino researcher noted “I have been involved in the women’s movement as a researcher for many years, and I am ashamed to say that I have never really thought about women with disability”. Initially, some experienced academic researchers were resistant to the idea of including co‐researchers with disabilities in the enumerator team for the household survey, feeling the presence of people with different types of impairment might hinder the process.. However, as one observed “It has been a steep learning curve for me, I never worked alongside women with disability before. I mean I was really surprised, they can really do it”. Our whole team came to see women as disabilities as capable, and as women whose SRH rights were threatened. This reinforced commitment to the program itself, and to working alongside women with disabilities in their efforts to advocate for their rights.

### Uneven Distribution and Sustainability of Benefits

While there were demonstrable benefits arising from our disability‐inclusive approach, these benefits were not distributed evenly among the research team members and particularly among the different women with disabilities involved. For example, for some of the co‐researchers and PAG participants, the intervention created a range of new opportunities to participate in their communities and contribute to disability advocacy. However, for other women with disabilities these opportunities were fewer and were undermined by family responsibilities, geographical location, social isolation, and the limited availability of the (often economic) resources required to sustain engagement over time. In some instances, the intersection of women’s impairments with particularly difficult life circumstances made ongoing participation in activities that emerged out of the W‐DARE program impossible. For others, acting on the knowledge, skills, and confidence they had gained through W‐DARE brought about new challenges—for example, some women who learned more about their rights through the PAGs decided to take action to leave their violent partners. This gave rise to new struggles because of the limits to social protection in the Philippines, and the lack of the social and legal services women with disabilities and their children require in such circumstances. These challenges further eroded the equitable and sustainable distribution of benefits from the program.

It is well established that women with different types of disabilities experience distinct barriers to accessing SRH and violence prevention information and services (Dowse et al., 2016; UNFPA, 2018). Therefore, it was important that W‐DARE included, and addressed the interests and needs of, women with diverse disabilities. The questions of who represents communities in CBPR projects, and which community members do and do not have opportunities to participate, are also well‐established ethical and practical concerns for researchers (Kwan & Walsh, 2018; Minkler, 2005). The representatives of the two DPOs that were formal W‐DARE research partners were able to ensure women with different types of disabilities were included as co‐researchers and participants. Women who had vision impairment and mobility impairments and who were deaf were actively involved throughout. However, the DPOs were less representative of (and had poorer connections to) women with other types of disabilities, particularly women with intellectual and psychosocial disabilities. As this became apparent, the W‐DARE team engaged and trained an activist with psychosocial disability to undertake dedicated research with other women with psychosocial disabilities, and conducted interviews with and developed a PAG intervention for parents of women with intellectual disabilities. However, our reflections suggest the benefits of the research process were less significant for these two groups. This is an important limitation to note given the high levels of discrimination and violence experienced by women with psychosocial and intellectual disabilities. As a research team, our ability to be genuinely and meaningfully inclusive of all women with all types of disabilities throughout W‐DARE was undermined by our limited expertise in working effectively with particular groups, resource constraints, and by the fact that DPOs themselves can also be exclusionary spaces for some women with disabilities.

In addition to the uneven distribution of the benefits of a disability‐inclusive approach to CBPR, our reflections highlight the very real challenge to the sustainability of any benefits that may arise. Some women with disabilities were able to draw upon the capacities they developed to realize ongoing opportunities and make improvements to their life circumstances. However, evaluation of the PAG intervention (Devine et al., 2017), and our reflections upon change some years after the W‐DARE program formally ended, suggests that this was not the case for all the women with disabilities we worked with or for all other members of the research team. The benefits arising through a participatory research process can be fragile and easily undermined in contexts of poverty, political turmoil, and structural violence.

One of the strategies used during the W‐DARE program to increase the sustainability of benefits and achievements was to engage with relevant government departments (particularly health, and social welfare and development) at national, provincial, and local levels. This was partially successful, with government service providers trained and subsequently some government facilities modified to increase physical accessibility. However, turnover of relevant personnel highlights the need for training to be sustained. It also points to the importance of including engineers and others responsible for health facility standards, construction, and accreditation in efforts to ensure that health facilities (current and planned) are fully accessible. W‐DARE initiated work with government around disaggregation of data to enable service providers to monitor uptake and use of services by people with disabilities. However, collecting and using data on disability is complex and also requires sustained intervention over time. In the lifetime of the program, we were unable to achieve the system wide change necessary for effective disability data collection and disaggregation. This highlights the limitations to what can be achieved by any one time bound research project, even if well‐resourced and conducted over a number years, emphasizing the need for sustained engagement to bring about structural change.

### The Material Costs of Inclusive Research

Facilitating the genuine and meaningful involvement of women with different types of disabilities in CBPR requires significant planning, and for researchers to include additional resources in budgets and timelines. These resources are required to address accessibility barriers by hiring wheelchair accessible training venues, providing accessible transport throughout project activities, providing sign language interpreters, covering costs of support people accompanying participants, distributing all project materials in accessible formats and so on. As noted by one of the co‐researchers:

Providing an accessible environment means increasing costs. Like if you need to provide interpreters. You need to pay the interpreters or the personal assistant if there is a need for [a participant] to get one. And for the participants, there is a [need for a] strategy to make sure that they can get there also and get back home.

Accessibility is a challenge for disability‐inclusive research in most contexts, but in the areas W‐DARE was working in was compounded by the poverty experienced by many people with disabilities. Prior to their participation in the groups, some of the PAG participants had barely left their houses in hilly informal settlements or in rural areas because of a lack of assistive devices, the lack of accessible transport, and the age and health of their family members. Some women with hearing impairments had never had the opportunity to learn Filipino Sign Language, and so could only communicate through gestural signs not well understood by anyone other than family members. As many of the women with disabilities involved in W‐DARE had never participated in research before, the extent of support and capacity building that was required for their meaningful participation was substantial. And as with any action research project, forecasting the cost of activities that are emergent from early data collection (i.e., unknown when the project starts) was challenging. A genuine commitment to disability inclusion in community‐based research projects requires researchers to anticipate these potential costs and ensure that contingency funding is preserved to prioritize accessibility.

### Distribution of Personal and Political Costs

In addition to the material costs associated with maximizing inclusion, our reflections highlight the personal and political costs associated with disability‐inclusive community‐based participatory research—particularly research on sensitive and potentially distressing topics such as SRH and violence. Many of our team, both women with disabilities and team members without disabilities, reported emotional and psychological “costs” associated with W‐DARE. Recognizing and addressing these is important within an ethical and analytical framework of reflexive solidarity (Davis & Vaughan, 2018; Vaughan et al., 2019).

Women with disabilities who were involved in data collection described the toll this had on them. The work was often physically fatiguing, but it was the emotional impact that co‐researchers felt most keenly. W‐DARE co‐researchers described the impact of hearing confronting stories from research participants as both distressing and as leading them to reflect on their own life experiences. Co‐researchers described how challenging it was to listen to, and sit with, difficult stories of violence and abuse:

We had this participant who shared her bad experience because she had been abused… I wanted to help her but when I got home I was so overwhelmed and confused. I kept thinking about her. So I got easily involved, emotionally involved.

Selection criteria for the co‐researcher role and the DPO networks through which co‐researchers were recruited meant that in comparison to many of the participants in interviews and the PAGs, co‐researchers often experienced relative privilege and opportunity. Some co‐researchers were unaware of the details of the desperate circumstances in which many other women with disabilities lived in the Philippines, and found this be highly confronting. To process what they were learning, W‐DARE co‐researchers relied on support from the program team and from each other, highlighting the importance of planning for and resourcing opportunities for debriefing.

While all members of the W‐DARE team were emotionally affected by the stories of the women we were working with, this “cost” was not evenly distributed and it was the co‐researchers who were most affected. Co‐researchers could empathize with women with disabilities on the basis of impairment and identity, shared experiences of discrimination, and barriers to the community. While this empathy resulted in high quality research data and was highly valued by participants, the emotional labor involved needs to be recognized. Practical solidarity between researchers with and without disabilities in this instance involves anticipating and planning for these costs, and working to address the impact through debriefing, carefully managing co‐researcher workloads, fostering intra‐team bonds through social events, and clear processes for minimizing and managing vicarious trauma.

In the context of the Philippines, research on SRH is not only sensitive and potentially traumatizing, but is highly political. This was particularly true over the period that W‐DARE was implemented, 2013–2016. The RH Law was decades in the making, with the advocacy and debate leading up to the final passing of the law in 2014, highly polarized. Debate around SRH issues at this time—in particular relating to access to sexual health education, contraception and abortion—was active in the media, in churches throughout the country, in the local government agencies who would be required to implement the law, and in the community. Members of the W‐DARE team, including team members from the women’s movement and from DPOs, came to the program with different positions in relation to the law. One of our first tasks as a research team was to listen to people’s different political and ideological positions, recognize areas where personal views were in conflict but also find areas of consensus. As noted by one of the Filipino researchers, our community‐based participatory approach facilitated this:

In the recent workshop that we just had, people became friends. In that forum we don’t talk about differences, we talk about how we can work together. To me that is an accomplishment already.

The costs associated with working through political and ideological difference were disproportionately born by the co‐researchers and other members of the research team based in the Philippines, but the benefits of this dialogical work were substantial. Team members who had a long history in the women’s movement in the Philippines as activist‐researchers and activist‐health practitioners informally mentored women with disabilities who were growing in their capacity as disability activists. Women with diverging ideological views about some aspects of SRH nonetheless stood together in highlighting the rights of women with disabilities at the Philippines Human Rights Commission, and in subsequent advocacy efforts. This advocacy and activism has continued well after W‐DARE finished, suggesting emergent solidarity between the feminist and disability movements in the Philippines. The work involved in sustaining this emergent political solidarity has been led by researchers, women’s health organizations and women with disabilities, and continues despite the considerable changes in the national political landscape over recent years.

Women with disabilities involved in the program, as partners, co‐researchers, and PAG participants were intensely interested in learning more about sex, sexuality, SRH, and their rights—information that had often been denied them because of their disability. The PAGs created an environment of learning but also one of friendship, where previously isolated women with disabilities were able to support each other in relation to what they were learning about SRH and rights. In solidarity, women with disabilities supported each other to take action in relation to seeking justice for abuse they had experienced. However, the barriers to justice for women with disabilities in the Philippines are enormous. For co‐researchers, participants, and researchers, pushing for justice on behalf of themselves and women who they were acting in solidarity with had a considerable financial, emotional, and social toll. This is likely to be the case for other research teams working in similar settings, where barriers to women with disabilities claiming their rights are political and entrenched in the structures of society.

### Sustaining Action after the Research

Toward the end of the W‐DARE program, one of our team based in the Philippines asked “we have built a good experience around W‐DARE and we would like to continue doing it, but how? And where? And when?” A framework of solidarity in action requires that the burden of responsibility for action does not sit on the shoulders of community members alone; rather, that responsibility for sustaining momentum for change should be shared between all members of the research team and research stakeholders. However, a number of factors undermined sharing of this responsibility in our experience.

As outlined earlier in our reflections, W‐DARE came about because of a fortunate co‐occurrence of an expressed research need and the availability of funding. When this funding opportunity ceased with a change in the Australian government’s development priorities, it became very difficult for Australian members of the team to provide ongoing support to our Filipino colleagues. Australian researchers have tried to maintain connection and provide support at a distance, but realize that the impact of these efforts is limited. Reflecting on the program as a team revealed that Australian researchers looked back on it with a mixture of pride in what was achieved, but also feelings of guilt, anxiety, and regret at having “started something but now not being able to be much use to our friends and colleagues in their ongoing struggles”. There were echoes of these mixed feelings in the reflections of our Filipino colleagues, with one noting that the impact of the W‐DARE activities on women with disabilities was like *“*we took a bottle of water, shook it up and created bubbles, and now we have just left it alone. But the bubbles do not disappear straight away.” Another Philippines‐based colleague noted that Australian team members have been supportive of Filipino colleagues in applying for further funding to sustain action arising from W‐DARE, but that the negative impact of these funding applications not being successful is not evenly shared: “they can afford not to do another project, or do this again here, because down the road they are all [university] staff, and they get paid. Meaning that regardless of whether they want to do this, there is still work for them.”

In the context of a project involving international collaboration, our experience has been that responsibility for sustaining action has fallen largely to local researchers, partners in the health sector, and to women with disabilities themselves. The strengthened relationships between local researchers, partners, and women with disabilities suggest that bonds of solidarity have emerged from the CBPR, but the benefits of these relations are precarious and the costs unevenly shared. One Philippines‐based researcher noted that a drawback of our participatory approach is that:

We have just raised expectations because you know, when they go back home it is about finding a job, it is about paying for the bills, it is about sort of the discrimination that they face. And unless they have empowerment in terms of their own livelihood, it’s business as usual… my personal challenge is how to create enough opportunities so that everybody gets a piece of the pie.

The solidarity between Filipino researchers and women with disabilities that developed during the program has resulted in two researchers, in particular, working tremendously hard in the years after the program to support women with disabilities, mentoring and identifying opportunities for them. These local researchers have collaborated with women with disabilities and DPOs to write grant applications to continue their advocacy and to set up their own organizations, connecting them with funding bodies and local government officials. One of the co‐researchers notes that working to sustain the gains of the PAG intervention, in particular, is important because of the barriers experienced by PAG participants themselves to conducting advocacy:

Whether that be through another grant or another project or another donor, it is just that we must carry that through because we have built enough mass and momentum. It is a pity not to do anything about it… Some PAG participants really look at PAG as a way to go to other places and you know, be involved… the absence of activities will really tie them up again in their houses.

Reflection on the W‐DARE program some years after implementation formally ended suggests ongoing impacts of the solidarity developed through the research process. It also suggests a degree of reflexive anxiety among the team about the role of (particularly internationally funded) CBPR projects initiating change in circumstances of prejudice, poverty, and substantial barriers to the community for women with disabilities. While the W‐DARE program successfully helped to strengthen the supply of, and demand for, SRH information and services for women with disabilities at the two research sites, local researchers note that without ongoing funding, these gains cannot be sustained. As one Filipino researcher notes:

We have this culture of ‘ningas kugon’… Ningas kugon is like grass, when you set it on fire it doesn’t blaze but it dies down peacefully. What will happen is that now that we are very active in building empowerment for disabled women. But if we are not able to sustain it, if we would cut it short, I think that It [their empowerment] will fall, even if they are eager. Because, the funding agencies even for this year or for two years they are into supporting the PWD [people with disabilities] community and then after two years they would divert their support to other projects.

## Discussion

Analysis of the W‐DARE story confirms that there are important benefits to a community‐based participatory approach to research and that these benefits can be shared by women with disabilities if research teams are gender‐sensitive and disability‐inclusive. The benefits of disability‐inclusive CBPR can be realized even in settings of poverty, prejudice, and challenging political circumstances. However, there are costs and challenges inherent in such an approach, disproportionately born by co‐researchers when exploring sensitive topics with peers (Evans, 2016; Vaughan et al., 2019). Co‐researchers can work with members of their communities to generate rich, impactful data about difficult issues, but this can also lead to co‐researchers experiencing vicarious trauma and distress (Burke, le May, Kébé, Flink, & van Reeuwijk, 2018). Our experience highlights the importance of recognizing this risk and planning and resourcing strategies to support all members of a research team, but in particular co‐researchers from the community. University‐based researchers taking active steps toward cost‐sharing by supporting community‐based researchers throughout and beyond a program of research are a practical demonstration of solidarity (Davis & Vaughan, 2018).

The W‐DARE program also highlights challenges that are particular to international collaborations between researchers from high‐income countries and those based in low‐ and middle‐income countries. Programs inevitably come to an end, but relationships and responsibilities do not. Reflecting on the program raised unanswered questions for the Australian researchers about their ongoing role. How do researchers based in high‐income countries sustain support to colleagues working in particularly difficult contexts? Is it right that support should be sustained, or does this risk undermining emergent leadership from local actors? Would an intentional focus on solidarity building—and therefore on cost‐sharing—through research mitigate some of the questions left unanswered by our reflections on W‐DARE? Our analysis of the W‐DARE story suggests that reflexive solidarity (Davis & Vaughan, 2018) may be a useful framework to inform the design and implementation of community‐based research projects in settings of poverty, prejudice, and inequality, and when research is exploring particularly sensitive topics.

A limitation of our process in writing this paper is that we were unable to go back to all members of the W‐DARE team to draw on their reflections on the program, our disability‐inclusive community‐based participatory research approach, and the unanswered questions raised above. This reflects some of the challenges inherent in any CBPR project, but that are perhaps amplified when this is undertaken by an international collaboration—afterward some people will continue to be in a position to reflect on the process, contribute to academic papers and discuss theoretical frameworks such as reflective solidarity; some people with be busy working to improve the lives of women with disabilities, with limited time and energy for such discussion; and some people will just want to get on with their lives.

## Conclusion

Ensuring that CBPR approaches are disability‐inclusive can contribute to the generation of high quality knowledge about the health inequalities experienced by people with disabilities. Disability‐inclusive research can also contribute to the impact of research, in relation to the social and structural change required to redress inequalities, but also in relation to the capacities and attitudes of research teams. However, undertaking research with people with disabilities about the health inequalities and violence many experience comes with costs that are disproportionately born by co‐researchers with disability and local allies. A framework of reflexive solidarity may strengthen research teams’ ability to recognize and more equitably distribute these costs throughout a program of CBPR.
